# Designing flows to resolve human and environmental water needs in a dam-regulated river

**DOI:** 10.1038/s41467-017-02226-4

**Published:** 2017-12-18

**Authors:** William Chen, Julian D. Olden

**Affiliations:** 1 Quantitative Ecology and Resource Management Program, University of Washington, Seattle, WA 98195 USA; 2 School of Aquatic and Fishery Sciences, University of Washington, Seattle, WA 98195 USA

## Abstract

Navigating trade-offs between meeting societal water needs and supporting functioning ecosystems is integral to river management policy. Emerging frameworks provide the opportunity to consider multiple river uses explicitly, but balancing multiple priorities remains challenging. Here we quantify relationships between hydrologic regimes and the abundance of multiple native and nonnative fish species over 18 years in a large, dryland river basin in southwestern United States. These models were incorporated into a multi-objective optimization framework to design dam operation releases that balance human water needs with the dual conservation targets of benefiting native fishes while disadvantaging nonnative fishes. Predicted designer flow prescriptions indicate significant opportunities to favor native over nonnative fishes while rarely, if ever, encroaching on human water needs. The predicted benefits surpass those generated by natural flow mimicry, and were retained across periods of heightened drought. We provide a quantitative illustration of theoretical predictions that designer flows can offer multiple ecological and societal benefits in human-altered rivers.

## Abstract (web summary)

Human and environmental water needs can come into conflict in dam-regulated river systems. Here, Chen and Olden investigate the potential for the use of fish–flow modeling to make recommendations for the management of native and nonnative fish species whilst providing water for society.

## Introduction

Human societies are grappling with the need to supply reliable and affordable water to growing populations, while at the same time not degrading freshwater ecosystems nor disrupting important ecosystem goods and services. Climate change is intensifying this challenge as droughts increase in both frequency and severity in many parts of the world, leading to greater risk of water supply deficits^1, 2^. Innovative strategies are now needed to account for, and assess trade-offs among, multiple potential river uses, taking into consideration the need for water security and the protection of critical ecosystem functions^3, 4^.

One of the most promising approaches to integrating human uses into the larger scope of ecological sustainability is the concept of environmental flows, or the provision of water within rivers to support positive ecological outcomes while maintaining the water needs of human society^5^. Recent decades have witnessed significant advances in the science underpinning environmental flow management, particularly in relation to prescribing water releases from large dams^6^, which now number in the tens of thousands globally, and growing^7^. These efforts, as well as most traditional water management practices, are founded on the fundamental principle that native plants and animals are adapted to natural (unaltered) flow regimes and will, therefore, benefit from dam operations that seek to emulate historical flow conditions^8, 9^. Indeed, streamflow alteration is a primary threat to freshwater ecosystems^10^. However, the human enterprise has already drastically changed how hydrology regulates riverine processes^11, 12^, thus raising the question of whether natural flow mimicry remains the most appropriate management goal for conserving freshwater biodiversity and ensuring functioning ecosystems.

The designer flow concept is an emerging paradigm to address the challenge of environmental flow management in human-altered rivers. Extensive reliance on rivers to produce hydropower, reduce flood risk, and store water for consumptive use cannot be avoided; therefore, the traditional approach where natural flow regimes are the target for environmental flow management may only be feasible for the least-regulated ecosystems^13^. Building from previous advancements in “holistic” approaches to water management^6^, the designer flow concept seeks to define the hydrologic conditions—which may deviate from natural flow conditions—that promote key ecosystem processes or biological outcomes of interest while navigating the increasingly competing, societal demands for water and flow^13–15^. Although dams alter the natural flow of rivers and threaten freshwater biodiversity, they also provide the prospect to design flows through their downstream release of water. Thus, designer flows have the potential to support freshwater conservation goals by mitigating dam-related impacts, while also striving to provide multiple social and economic benefits.

Designing environmental flows for rivers is hampered by the lack of robust models that explicitly account for multiple human and ecosystem needs^4, 16^, particularly with respect to contrasting ecological targets. Water management practices that balance multiple ecological objectives are challenging to achieve. Dams and their regulation of downstream hydrology have allowed many invasive species to thrive in rivers where natural flow regimes previously hindered their establishment^17^. Invasive fish species are a leading threat in freshwater ecosystems^18^, and thus are an increasingly important consideration when defining dam-related environmental flow prescriptions. Although ecological knowledge suggests that a natural flow regime should simultaneously benefit native species and disadvantage nonnative species^8^, the reality is that fishes show a variety of responses to flow regimes that do not necessarily align with their status of origin^19, 20^. Thus, efforts to manage river flows to mimic historical flow conditions may unintentionally assist nonnative species and even fail to achieve the full benefit to native species^8, 9, 17^.

**Fig. 1:**
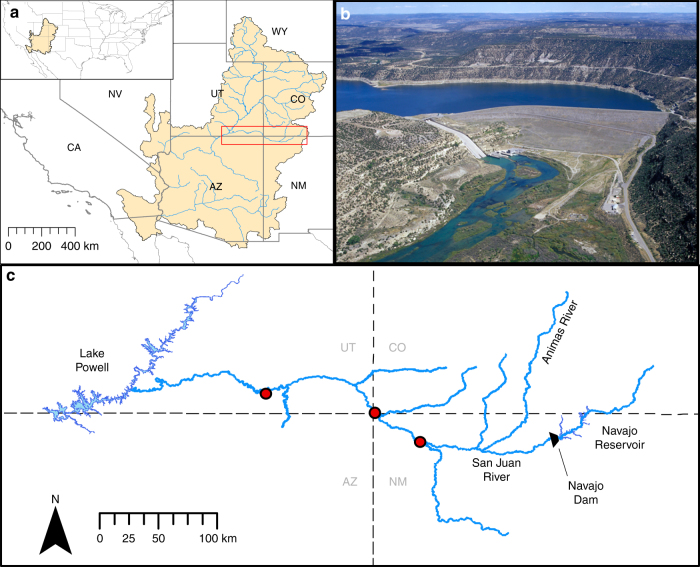
Map of study region. **a** The San Juan River Basin (red box) is a major tributary of the Colorado River Basin, located in southwestern United States. **b** The Navajo Dam (height, 123 m) is an earthen dam on the San Juan River that creates the Navajo Reservoir. **c** Our case study focused on the region from Navajo Reservoir (river kilometer 360) to Lake Powell (river kilometer 0). Red circles indicate the USGS flow gage stations used in the study. Photo credit: United States Bureau of Reclamation

Here we apply multi-objective optimization to narrow the knowledge gap between the designer flow concept and the science needed to support this approach for sustainable water management when confronted with multiple ecological considerations. Using a large, dam-regulated, dryland river in southwestern United States (Fig. 1) as an epitome of water-resource challenges in a changing climate, we forecast the trade-offs among allocating water for three objectives: (1) ensure sufficient water for agricultural, domestic, and industrial supply (hereafter, termed human water needs), (2) benefit populations of native fishes, and (3) inhibit populations of nonnative fishes. We first examine the contemporary relationship between annual hydrographs and the relative abundance of multiple native and nonnative fishes using functional regression models. Second, we incorporate the resultant flow–ecology relationships along with a dam operations model into a multi-objective optimization framework. Finally, we use this optimization model to identify facets of the designer flow regime that are predicted to efficiently meet both human and ecosystem water needs and support explicit and actionable prescriptions for daily dam releases. We consider multiple climatic scenarios that encompass the range of water availability in the region (including hydrologic drought conditions), and we evaluate the potential of designer flows to promote native fish biodiversity in comparison to attempts to mimic natural flow regimes.

## Results

### Quantifying fish–flow relationships

Functional regression models were constructed to quantify associations between daily discharge and autumn abundances of native and nonnative fishes in the San Juan River. Fish–flow models explained, on average, 35% of the variance in fish densities as a function of discharge across years and locations (Supplementary Table 1), and fish responses to flow conditions in the San Juan River were broadly similar to modeled associations in other major river basins in the region (Supplementary Fig. 1). Predictions from the fish–flow models provided the basis for the multi-objective optimization described below.

### Designer flows support human and ecological water needs

**Fig. 2:**
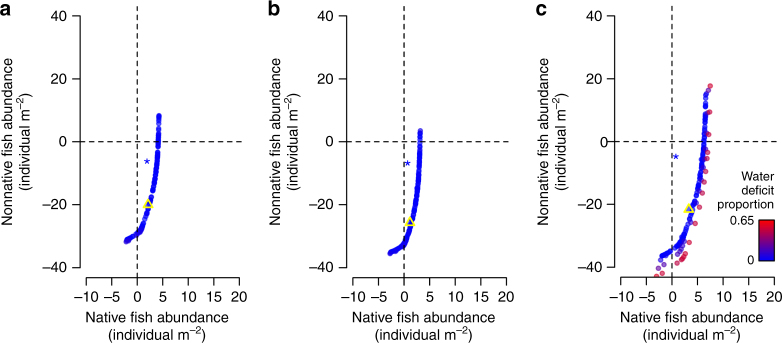
Pareto frontiers depicting optimal flow designs for Navajo Dam. Designer flows differentially prioritized human water needs, average native fish abundance gains, and average nonnative fish abundance losses during the **a** wet (high-flow) period, **b** normal (average-flow), and **c** dry (low-flow) period. Predicted effects on abundances (individual m^−2^) are on the log-scale averaged across all years, river reaches, and species within each scenario. Each point represents a different flow design. The triangle symbol in each panel designates sample flow design used for discussion of designer flow approach in main text. The star symbol in each panel indicates the outcomes of hypothetical natural flow mimicry with no deficit to human water needs

Designer flows consistently outperformed natural flow mimicry in simultaneously meeting human water needs and promoting ecological goals (Fig. 2). For illustrative purposes, we focused on one of several flow designs that heavily prioritized human water security and concurrently sought to balance the objectives of native abundance gains versus nonnative abundance losses equally (i.e., minimal human water deficit, moderate native abundance gains, and moderate nonnative abundance losses). Most striking was that designer flows were predicted to lead to over 200% greater nonnative abundance losses when compared to natural flow mimicry (Table 1). In addition, designer flows were always predicted to benefit native species, whereas flow prescriptions that mimicked natural flow regimes led to small losses in native fish abundance during periods of average or low water availability and only small gains in high-flow years.

**Table 1:** Predicted average native and nonnative abundance gains and losses.

Water availability	Designer flows		Natural flow mimicry	
	Native	Nonnative	Native	Nonnative
Wet (high-flow)	2.11	−20.00	0.29	−9.27
Normal	1.13	-25.67	-0.42	−9.12
Dry (low-flow)	3.29	−21.61	-0.26	−6.77

Gains (+) and losses (−) resulting from designer flows (left) versus natural flow mimicry (right) for three climatic scenarios of decreasing river discharge (flow). Abundances (individual m^−2^) are presented on the log-scale averaged across all years, river reaches, and species within each scenario. Designer flows heavily prioritized human water needs and equally balanced native abundance gains and nonnative abundance losses (90%/5%/5% respective priority split)

The benefit of designer flows for favoring native over nonnative fishes further surpassed those generated by natural flow mimicry during a period of heightened hydrological drought. Predicted improvement to native fish abundances from our example designer flows versus natural flow mimicry grew by nearly 100% when comparing the low-flow, dry (below-average annual river discharge) climate scenario to the high-flow, and wet (above-average annual river discharge) scenario (Table 1). Meanwhile, improvement in nonnative abundance losses for this same comparison increased by nearly 40%.

These comparisons represent but one set of priorities for flow designs. Our optimization procedure identified many possible flow prescriptions as leading to simultaneous benefits to fish conservation goals (i.e., native fish abundance gains, nonnative fish abundance losses) and to society by meeting water needs for agricultural, domestic, and industrial use, even during drought conditions (Fig. 2). Hundreds of different flow designs rarely, if ever, encroached on human water needs, and perhaps most importantly, opportunities for achieving multiple benefits did not disappear during periods of limited water availability. Only the dry climatic scenario saw flow designs that resulted in major deficits to human water needs, and then only when disproportionately prioritizing ecological objectives.

### Designer flows highlight fish management trade-offs

**Fig. 3:**
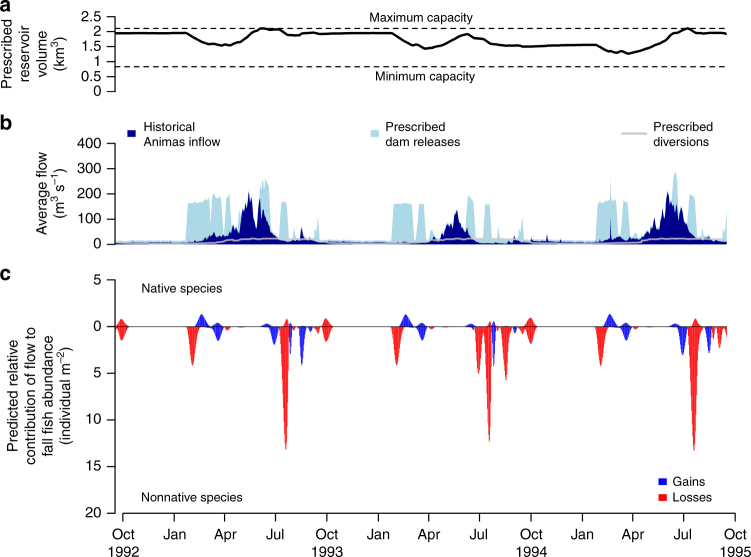
Designer flow prescription balancing human and ecological objectives from 1992 to 1995 during the wet (high-flow) period. Designer flow prescription indicated by triangle symbol in Fig. 2a. The depicted designer flow heavily prioritized human water needs and focused equally on native species abundance gains and nonnative species abundance losses (90%/5%/5% respective priority split). **a** Prescribed reservoir volume; **b** Cumulative plot of historical Animas River inflow (dark blue) and prescribed Navajo Dam releases (light blue), with prescribed water diversions downstream of Navajo Dam overlaid (gray); **c** Predicted relative abundance gains (blue) or losses (red) of native (above zero axis) and nonnative (below zero axis) fishes in response to flow designs (individual m^−2^ on log scale)

**Fig. 4:**
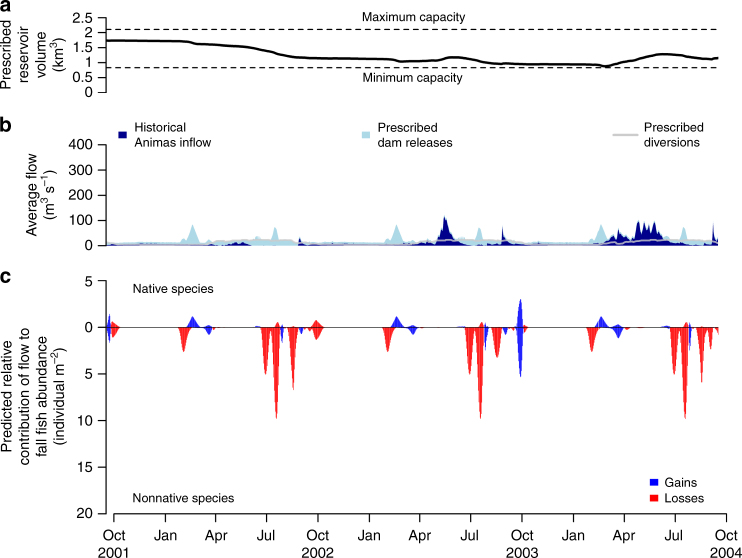
Designer flow prescription balancing human and ecological objectives from 2001 to 2004 during the dry (low-flow) period. Designer flow prescription indicated by triangle symbol in Fig. 2a. The depicted designer flow heavily prioritized human water needs and focused equally on native species abundance gains and nonnative species abundance losses (90%/5%/5% respective priority split). **a** Prescribed reservoir volume; **b** Cumulative plot of historical Animas River inflow (dark blue) and prescribed Navajo Dam releases (light blue), with prescribed water diversions downstream of Navajo Dam overlaid (gray); **c** Predicted relative abundance gains (blue) or losses (red) of native (above zero axis) and nonnative (below zero axis) fishes in response to flow designs (individual/m^−2^ on log scale)

Designer flows capitalize on empirically based, predicted differences between native and nonnative responses to river flow (Supplementary Fig. 2). In general, high late-winter (February) flows simultaneously benefited native flannelmouth sucker (*Catostomus latipinnis*) and speckled dace (*Rhinichthys osculus*), while disadvantaging nonnative red shiner (*Cyprinella lutrensis*), fathead minnow (*Pimephales promelas*), and channel catfish (*Ictalurus punctatus*). On the other hand, native and nonnative fishes both benefited from increased mid-spring (April) flow magnitudes, and both responded negatively to increased mid-autumn (October) flow magnitudes. Higher flow releases late-summer demonstrated a strong negative influence on nonnative species, with negligible effects on native species. Consequently, our predictions suggest that flow designs that favor native over nonnative fishes in the San Juan River could involve large dam releases in late-winter to benefit native species at the expense of nonnative species, carefully managed releases in the mid- and late-spring fish spawning months to consider relative benefits to both native and nonnative species, and additional releases in the late-summer low-flow period to the detriment of nonnative species (Figs. 3 and 4). Flow designs also tracked changing water availability, reserving water when unregulated Animas River flow could readily provide for societal water diversions in order to maximize ecological benefit of dam releases at other times of the year.

**Fig. 5:**
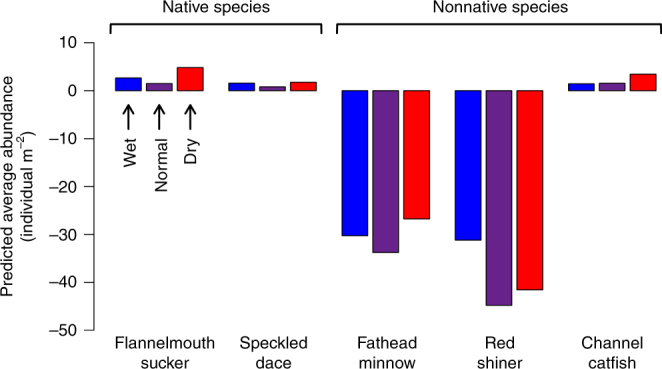
Fish species abundance gains or losses according to the designer flow prescription. Designer flow prescription indicated by triangle symbols in Fig. 2.Values are averaged across three-year periods under wet (blue fill), normal (purple fill), and dry (red fill) climatic scenarios. Species responses are based on flow designs that heavily prioritized human water needs and gave equal consideration to native fish benefit (*Rhinichthys osculus* and *Catostomus latipinnis*) and nonnative fish detriment (*Cyprinella lutrensis*, *Pimephales promelas*, and *Ictalurus punctatus*)

Despite these overall trends in flow designs, we found individual predicted species’ responses to flow designs did not necessarily align along nativity groupings (Fig. 5). Across climatic scenarios, flow designs simultaneously benefited both native fish species, while greatly decreasing abundances of the two small-bodied, nonnative competitors—red shiner (*Cyprinella lutrensis*) and fathead minnow (*Pimephales promelas*). By contrast, the large-bodied, nonnative channel catfish (*Ictalurus punctatus*) consistently showed weak positive responses to optimal flow designs, thus demonstrating the unavoidable trade-offs associated with managing dam releases for entire assemblages of species.

## Discussion

The past decade has seen considerable empirical and methodological advancements in understanding the functional flows required to support ecosystems (e.g., refs. ^14, 21,]^), thus providing the foundation for the multi-objective optimization framework implemented here. We found that designer flows could provide greater potential for disadvantaging nonnative species compared to natural flow mimicry, and that dam release schedules informed solely by historical flow conditions may fail to achieve native species conservation goals fully (Table 1). This trend in native fish responses is not predicted by the ecological literature^8, 9^, and instead likely suggests that the response of native fishes to flow regimes within our study system may be mediated by the presence of nonnative fishes or influenced by other environmental drivers^12^. Previous studies have shown the potential of introduced fishes to affect native fishes’ behavior and habitat use^22^, which can modify native flow responses^23^. Building on theoretical frameworks that propose greater opportunities for ecosystem sustainability resulting from designer flows^13, 14^, we provide a quantitative illustration for these predictions in a human-altered river.

A suite of dam operation strategies that simultaneously met both societal and environmental water needs underscored the potential benefits provided by designer flows (Fig. 2). Ensuring human water security versus supporting ecosystems services via natural hydrology have long been considered conflicting objectives in water-resource management^24^, a perspective that has been reinforced by previous optimization studies^25, 26^. Most multi-objective optimization studies have focused on balancing human water needs with the goal of releasing environmental flows to mimic a natural flow regime or of solely benefiting native fishes^27, 28^. By contrast, our approach looks beyond the natural flow paradigm and considers multiple native and nonnative species explicitly in the design of environmental flows. The notions that societal water-use inherently precludes the ability to achieve positive conservation outcomes, and that the severity of trade-offs between these two goals only intensifies as water availability continues to decrease, is pervasive among scientists, practitioners, and the public^4, 29^. Our results suggest that these trade-offs can be overcome through multi-objective optimization and careful planning.

Despite mounting pleas and accumulating science to define environmental flows for freshwater ecosystems, previous efforts have overwhelmingly focused on single species or ecosystem surrogates for river restoration^30–32^, with little consideration of explicit targets for biological communities^33^. By incorporating multiple species associations with the entire hydrologic regime, we suggest that designer flows may be engineered to meet human water demands and take advantage of mismatches between native and nonnative species responses to flow. These mismatches create small, but powerful, windows of opportunity to allocate water for dam releases that deliver multiple ecological outcomes; in this case, supporting native species conservation and nonnative species control. Capitalizing on such opportunities are admittedly challenging and require interdisciplinary collaborations among researchers, engineers, watershed planners, and policy makers, just to name a few.

The sheer prevalence of nonnative fishes in dam-impacted rivers and the considerable similarities in life histories exhibited by native and nonnative fishes^20^ necessitate a multi-species approach to water management. Native and nonnative fishes did not always demonstrate contrasting responses to high flows (Figs. 3 and 4), which is indicative of ecological and flow-preference similarities^19^. Flow responses varied within nativity groupings, as seen with the channel catfish (*Ictalurus punctatus*), which responded to flow designs more similarly to native species compared to other nonnative species^19^. The fact that flow designs may have a net negative effect on nonnative fish communities, but that a spectrum of species-specific responses are possible, reinforces multi-faceted strategies to nonnative species management. For example, while populations of small-bodied fish species are infeasible to control physically in river systems, opportunities for active mechanical removal efforts for larger species exist^34^. Given that predation and competition from introduced species are major threats to native biodiversity and are exacerbated by human alteration of rivers^18^, it is critical to quantify the trade-offs inherent in environmental flow prescriptions that seek to disfavor multiple nonnative species.

Most dam release experiments—while founded on a robust understanding of species’ responses to natural flow conditions—have predominantly implemented only simple flow recommendations based on single flow events^33^. We found that isolated flow events often failed to simultaneously bolster native species and deter nonnative species (Fig. 3). Indeed, flood manipulations in other southwestern rivers of the United States have benefited some native fishes, but with limited to no effect on nonnative fishes^35, 36^. Increased flooding in the Murray–Darling River Basin, Australia, showed similar inconsistency in responses of native and nonnative fishes^37^. By contrast, environmental flow prescriptions that were motivated by multiple ecological processes created more opportunities for native fishes to flourish over nonnative fishes^32^. Evaluating trade-offs and informing flow management in human-altered rivers require identifying the manifold facets of the flow regime that support desired ecological structure and function^14^. Our study quantifies this knowledge and integrates regime-wide flow ecology for multiple species into environmental flow prescriptions for the river basin under study; this analytical approach is readily transferable to other river systems across the world and could be prioritized towards dams where benefits to river biodiversity are likely to be maximized^38^.

Environmental flow management is often overlooked during years with below-average flow because of the perceived scarcity of water available to meet ecological objectives after human demands have been satisfied. We assert that this represents a potential lost opportunity. Although low water availability creates challenges for prescribing the flood pulses that often form the basis of natural flow mimicry, our results demonstrate considerable scope for achieving ecological outcomes using designer flows of all magnitudes and timing. Specifically, hypothetical flow designs that prioritized dam releases in late-winter, late-summer, and mid-autumn were predicted to favor native over nonnative fish populations, even in drought conditions (Fig. 4). Here, flow designs depended on the unregulated inflow of water from the Animas River into the San Juan River. During high-flow years, Animas River inflow provided water for societal water diversions, whereas drought conditions placed greater weight on environmental flows relative to unregulated flow for meeting ecological and societal water needs. Low-flow hydrology is critical for fish movement, spawning, and recruitment^39^, and environmental flow management has shown some success in reversing the impact of human alteration on low-flow events^40^. Elevating the value of targeted environmental flow management under water scarcity will reveal new and unexpected opportunities for freshwater conservation in an increasingly drought-stricken future^41, 42^.

Quantitative models that support more diversified options for utilizing environmental flows to target multiple species and ecological processes provide exciting opportunities to tailor prescriptions for entire ecosystems. For example, experimental floods have successfully restored native riparian vegetation^43^, returned aquatic macroinvertebrate communities to pre-dam conditions^44^, and invoked food web responses in river ecosystems^45^. Moreover, environmental flows have the potential to reverse the detrimental effects of dams on riverine thermal regimes^12, 46^ and sediment transport^47^. A central challenge for the adoption of designer flows will be the explicit consideration of desired physical and biological outcomes, leading to a truly holistic or ecosystem approach. Not meeting this challenge will ultimately impede the translation of flow designs from theory to practice. Our study suggests how we might accomplish this integration via multi-objective optimization to inform dam operation strategies.

Considerable scope also exists for designing environmental flows that are informed by multiple social and economic objectives, while still supporting functioning freshwater ecosystems. For example, designer flows are equally pertinent to managing potential conflicts between hydropeaking operations and biodiversity conservation^48^, a particularly relevant challenge given the thousands of existing and new hydropower dams planned for construction around the world^49^. Optimizing flow designs around minimizing operational costs creates opportunities for evaluating the economic value of ecosystem goals and increasing potential ecological benefits per dollar spent^15^. Furthermore, dams provide opportunities to manage, and thus optimize, downstream water temperature regimes^12^, leading to discussions on whether dam operations can mitigate warming effects from climate change^50^. Finally, the modular nature of multi-objective optimization allows for evaluating additional societal dimensions, such as accounting for lake-level fluctuations and thermal structures that influence greenhouse gas (carbon dioxide and methane) emissions from reservoirs^51^.

The literature on flow–ecology relationships is now substantial^21^, but we recognize that predictions from our study are only as strong as the validity of the fish-ecology models^16^. For this reason, we openly acknowledge the following caveats. First, our designer flow predictions could be undermined if the modeled fish–flow associations revealed themselves to be inaccurate representations of reality or driven by spurious correlations. This is important to recognize because our models only explained modest amounts of variance in fish densities (Supplementary Table 1), and demonstrated similar, albeit variable, fish–flow relationships compared to other dryland river systems in the region (Supplemental Fig. 1). Second, because the designer flow approach relaxes the assumption of adhering to a natural flow regime, flow designs may fall outside the range of hydrological conditions used to inform the fish–flow models. Third, the interactive effects of streamflow, water quality, and physical habitat characteristics on ecological responses are widely recognized, but rarely incorporated (included here) into flow–ecology relationships^12^. Fourth, we leveraged recent statistical advances in functional data analysis to enable a more holistic characterization of the flow regime and its relation to fish species density^52^, yet we recognize that the ecological basis for flow prescriptions remains uncertain. For example, our models predicted that native fishes could be favored over nonnative fishes using designer flows prescribed on the scale of days and weeks, yet the manner in which fish species may truly respond to the accumulation of these flow events over the year requires additional consideration. In summary, whether it is broad flow-management frameworks^31^ or quantitative optimization approaches, the validity of environmental flow designs are inextricably linked to the robustness of underlying flow–ecology models. The present study is no exception. Greater scrutiny into flow–ecology associations, particularly accounting for non-linear variable relationships^53^, non-stationarity in hydro-climatic and ecological processes^54^, and variable flow responses across ecological metrics^55^, remain a critical research frontier.

How multiple benefits from designer environmental flows are realized across different groups in society depends on the physical and social structures, as well as the political economy from which access and entitlements to these benefits are mediated^4, 56^. Successful environmental flow implementation requires diverse and authentic stakeholder involvement to define and refine desired social and biological outcomes^4, 57^. We have illustrated how particular flow designs may best promote native fish conservation after accounting for human water needs, but these decisions ultimately depend on the shifting values that stakeholders place on a whole suite of competing objectives. These values are also likely to morph in response to changes in water availability, human water needs, and natural resource management goals^1, 15^. Region-wide planning and consensus building remains essential to achieving multiple benefits from environmental flow management, and there is no denying that many water-resource decisions are made in contentious contexts constrained by political, legal, social, and economic realities.

Despite the oft-cited headlines of mounting conflicts between human and ecosystem needs for water, we predict that such trade-offs can be creatively navigated and potentially avoided by using multi-objective optimization^58^. This approach allows for the discovery of efficient solutions that minimize conflict among competing water needs when trade-offs cannot be avoided. In human-altered rivers facing multiple stressors that include invasive species, mimicking natural flow regimes below dams may be just one of many options for conserving freshwater diversity. Designer flows have been suggested as a means to enable ecosystem design and support adaptation to environmental change, and here we provide a quantitative, albeit untested, illustration of how this emerging paradigm can be realized to ensure both societal and environmental benefits of dam operations. Further advancements in the development of multi-species functional flow–ecology models and the incorporation of environmental, social, and political stochasticity in flow design prescriptions will increase consensus on designer flows as the operating standard for human-altered rivers.

## Methods

### Study system

The San Juan River Basin (SJRB) is in an arid to semi-arid region of southwestern United States (Fig. 1). Regulated releases from Navajo Dam on the mainstem and unregulated inflow from the Animas River tributary largely determines its hydrology. The SJRB exhibits high mean daily spring discharge and low mean daily summer discharge (Supplementary Figs. 3 and 4), though the Navajo Dam has greatly decreased spring discharge magnitude and interannual summer discharge variation^36, 59^. This dynamic, snowmelt-fed system led us to consider multiple climatic scenarios: periods of wet high-flow, normal average-flow, and dry low-flow conditions. We used the Discrete Fast Fourier Transform to extract the seasonal component of logarithmic-transformed, normalized streamflow records at the USGS flow gage station near Four Corners, CO (USGS 09371010) from 1985 to 2014^60^. We then calculated the total annual deviation from this seasonal component to characterize water availability in each year (Supplementary Fig. 5). Based on this analysis, we identified three-year periods that represented: (1) positive anomalies indicating higher-than-average flow conditions (water years 1993–1995; water years start in October); (2) minimal anomalies indicating average flow conditions, (water years 1999–2001); and (3) negative anomalies indicating lower-than-average flow conditions (water years 2002–2004). A year of average flow conditions preceded each period. We used three-year periods to match general planning periods for Navajo Dam operation rules, though other planning periods could be investigated. These periods represent wet, normal, and dry flow conditions, respectively, and reflect historical differences in Navajo Dam water releases, inflow into the Navajo Reservoir, and inflow from the Animas River tributary (Supplementary Table 2).

The Navajo Reservoir primarily serves as a water storage facility for agricultural, domestic, and industrial use within the SJRB. The Navajo Indian Irrigation Project (NIIP) comprises the largest portion of the water needs in the SJRB, which draws water directly from the reservoir, while other water diversions occur at numerous locations below Navajo Reservoir. These diversions, which were similar across the three climatic scenarios, are concentrated in March through October, and peak during June and July. We modeled human water-use needs within the SJRB using United States Bureau of Reclamation (USBR) water depletion data (Supplementary Fig. 6). Although there are return flows from water diversions within SJRB, we made the conservative and simplifying assumption that return flows from river diversions were negligible relative to channel flow^61^.

The SJRB supports numerous native and nonnative fishes and is a critical stronghold for several threatened fish species^36^. Dam-related flow regime alterations have led to considerable spatial overlap of native and nonnative fish populations^62^. Management actively seeks to increase the abundance of desired native fishes, while concurrently depressing the populations of nonnative fishes^63^. We collated time series of native and nonnative fish abundances in San Juan River secondary channels (river kilometers 110–248) collected once a year between mid-September and mid-October (hereafter, “Fall”) from 1993 to 2010 per standardized shore seining protocols from the SJRB Recovery Implementation Program^36, 59^. Surveyors used a 2.2 m × 1.9 m × 3.0 mm mesh drag seine, made at least five hauls per secondary channel to sample all distinct habitats, with additional hauls for secondary channels with greater habitat diversity, and estimated the area of each haul. Fish surveys targeted a range of habitats (backwaters, pools, riffles, runs, and shoals) in 200-m long secondary channels (characterized as <25% of the main channel discharge). We focused on two native (*Rhinichthys osculus* speckled dace and *Catostomus latipinnis* flannelmouth sucker) and three nonnative (*Cyprinella lutrensis* red shiner, *Pimephales promelas* fathead minnow, and *Ictalurus punctatus* channel catfish) fish species. These species span a range of flow and mesohabitat preferences, are sufficiently abundant in the SJRB to be effectively sampled, and exhibit similar life histories to species that were not included in our study^19, 36^.

### Modeling fish–flow relationships

We used historical discharge data (in m^3^/s) from three USGS flow gaging stations to examine the daily influence of flow magnitude on focal species’ Fall abundance: Shiprock, NM (USGS 09368000), Four Corners, CO (USGS 09371010) and Mexican Hat, UT (USGS 09379500). Discharge was similar across all three points of the river, spanned a wide range of hydrologic conditions, and is representative of hydrologic conditions that persist in the San Juan River Basin after the construction of Navajo Dam (Supplementary Fig. 4). We calculated the density of focal species (individuals per m^2^ seined) within corresponding geomorphic reaches: Red Wash, NM to Shiprock, NM, river kilometers 211–248; Aneth, UT to Red Wash, NM, river kilometers 173-211; and Chinle Creek to Aneth, UT, river kilometers 110–173. By considering flow responses across three reaches, we minimized the risk that hydrological or species density idiosyncrasies would affect the analysis. Both densities and river discharges were logarithmic-transformed to reduce the influence of disproportionately large values in our analyses.

We implemented functional regression models to estimate the influence of local daily discharge on native and nonnative fish abundances throughout the SJRB. Functional data analysis is more appropriate than traditional regression analysis for data that take the form of functions rather than single values^64^, and is considered to provide a more holistic characterization of flow regimes than traditional hydrologic metric approaches to fish–flow relationships^52^. Rather than using hydrological summary statistics, functional regression uses annual hydrographs as the environmental covariate to predict fish abundances. The basic (discrete) form of the regression model is:

1 $$y_i = \beta _0 + \mathop {\sum }\limits_t \beta _1\left( t \right)f_i\left( t \right) + {\it{\epsilon }}_i,$$

where *t* spans the days of the year, *y*_*i*_ is the fish abundance in year *i*, *f*_*i*_(*t*) is the log-transformed flow magnitude in year *i* on day *t*, *β*_0_ is the average fish abundance, *β*_1_(*t*) is the regression coefficient indicating the daily influence of flow on fish abundance, and *ε*_*i*_ accounts for unexplained variation in fish abundance. We extended this approach by adopting a methodology—termed functional regression that is interpretable (FLiRTI)—that preserves the desired, smooth function estimation of the basic functional regression approach, but improves the interpretability of the estimate by constraining nonzero effects of predictor variables to only the most relevant parts of the function’s domain^65^. While we acknowledge that hydrograph idiosyncrasies could skew interpretations of flow–ecology relationships, such effects also manifest in hydrological statistics.

Using the FLiRTI approach, we determined the daily influence of antecedent flows (prior year) on annual Fall abundances of each species within each reach, assessed model fit via five iterations of sixfold cross-validation, and selected models that exhibited the smallest median cross-validated error, to avoid overfitting to our data^65^ (Supplementary Table 1). From these models, we obtained regression coefficients $\beta _{xr}(t)$ for species *x* in reach *r* on each day *t*, which we used to predict the cumulative effect of flow *M*_*xry*_ on a species *x* in reach *r* during year *y* given different hypothetical discharges $Q_{G,r}(y,t)$:

2 $$M_{xry} = \mathop {\sum }\limits_t \beta _{xr}(t)\log Q_{G,r}(y,t),$$

Each individual species’ responses to potential flow regimes were calculated by averaging *M*_*xry*_ across all reaches and years for that species.

We assessed the generality of the functional regression models by comparing fish–flow relationships of the SJRB to those derived for four other dryland rivers in southwestern United States—the Virgin River (Utah), Pecos River (New Mexico), Gila River (New Mexico), and San Pedro River (Arizona). Like the SJRB, extensive monitoring in these other rivers includes time series of annual surveys of fish species abundances and daily observations of streamflow at various sites^19, 60^. Functional regression models for 18 locations in the Virgin, Pecos, Gila, and San Pedro rivers were developed^66^ and the *β*_1_(*t*) regression coefficients from these models were compared to the SJRB fish–flow model.

### Efficient flow designs via multi-objective optimization

We modeled daily dam operations, human water-use diversions, and SJRB hydrology over each 3-year climatic scenario using mass-balance equations to elucidate daily dam water releases and water-use diversions that led to optimal flow designs. See Supplementary Table 3 for a summary of all parameters and variables used in the modeling framework, and see Supplementary Fig. 7 for a schematic of the study system.

We evaluated flow designs based on their efficiency at balancing trade-offs among three objectives: (1) minimizing the proportional deficit between prescribed water diversions and SJRB water needs, (2) maximizing SJRB native fish abundance gains, and (3) minimizing nonnative fish abundance gains. To quantify the degree to which a flow design satisfied human water-use needs in the SJRB, we calculated the total proportional deficit between the amount of water diverted $Q_{D,r}$ and the amount required $d_r$ overall reaches *r*, years *y*, and days *t*:

3 $${\rm{WD}} = \frac{{\mathop {\sum }\nolimits_{r,y,t} \left[ {d_r\left( {y,t} \right) - Q_{D,r}(y,t)} \right]}}{{\mathop {\sum }\nolimits_{r,y,t} d_r(y,t)}}.$$

Native and nonnative fish abundance gains (NF and NNF, respectively) were designated as the aggregated effects of flow estimated from functional regression *M*_*xry*_ averaged over each reach, year, and species within native and nonnative groupings.

To meet these objectives, we must decide the Navajo Dam water release rate $Q_R\left( {y,t} \right)$ on each day *t* of each year *y*. We must also decide the water withdrawal rate from Navajo Reservoir $Q_{D,0}\left( {y,t} \right)$, and the water diversion rate from each of our channel reaches: between Navajo Reservoir and Shiprock $Q_{D,1}\left( {y,t} \right)$, Shiprock and Four Corners $Q_{D,2}\left( {y,t} \right)$, and Four Corners and Mexican Hat $Q_{D,3}\left( {y,t} \right)$. These decisions are constrained by the physical and operational limits of Navajo Reservoir. The volume of water in the reservoir must be small enough to avoid overtopping the dam (*S*_max_) and large enough to ensure that the Navajo Indian Irrigation Project can draw water from the reservoir (*S*_min_):

4 $$S_{\rm{min}} \le S\left( {y,t} \right) \le S_{\rm{max}}.$$

We additionally constrained the reservoir volume on the first and last days of each simulation to be equivalent to actual, historical reservoir storage on the corresponding days. Alternatively, reservoir volume on the first day of each simulation could be constrained to be equal to that of the last day, but we deemed this constraint unrealistic during drought conditions. Dam releases$Q_R\left( {y,t} \right)$ from the reservoir are operatively limited by minimum ($Q_{R,\rm{min}}$) and maximum flows ($Q_{R,\rm{max}}$):

5 $$Q_{R,\rm{min}} \le Q_R\left( {y,t} \right) \le Q_{R,\rm{max}}.$$

In addition, daily dam releases cannot change by more than a limit $\delta _{R,\rm{max}}$:

6 $$|Q_R\left( {y,t + 1} \right) - Q_R\left( {y,t} \right)| \le \delta _{R,\rm{max}}.$$

Finally, we restrict diversions $Q_{D,r}\left( {y,t} \right)$ in each region *r* to be nonnegative and no more than the water demand for that region $d_r(y,t)$:

7 $$0 \le Q_{D,r}\left( {y,t} \right) \le d_r\left( {y,t} \right).$$

SJRB hydrology is subsequently constrained by our water diversion and dam release decisions, and vice versa. Water flows into the Navajo Reservoir from the San Juan River and its tributaries upstream of the Navajo Dam at a rate of $Q_{I,SJ}$; we considered direct precipitation on the reservoir surface and groundwater contributions to be negligible based on available evidence^67^. The main outflows from the Navajo Reservoir are releases from the Navajo Dam, $Q_R$, water diversions directly from the reservoir for the Navajo Indian Irrigation Project (NIIP), $Q_{D,0}$, and evaporative water loss from the reservoir, *E*. Consequently, the amount of water in the reservoir *S*(*y*, *t*) on day *t* of year *y* is a function of the amount of water stored on the previous day *t* − 1 and the inflows and outflows on the current day:

8 $$S\left( {y,t} \right) = S\left( {y,t - 1} \right) - E\left( {y,t} \right) + \gamma \left[ {Q_{I,SJ}\left( {y,t} \right) - Q_R\left( {y,t} \right) - Q_{D,0}\left( {y,t} \right)} \right],$$

where γ is a constant that extrapolates flow rates into daily volumes, assuming a constant flow within each day. The discharge at each USGS flow gaging station $Q_{G,r}\left( {y,t} \right)$ can then be predicted using mass-balance equations (flow into and out of a point must be equivalent):

9 $$Q_{G,1}\left( {y,t} \right) = Q_R\left( {y,t} \right) - Q_{D,1}\left( {y,t} \right) + Q_{I,A}\left( {y,t} \right),$$

10 $$Q_{G,2}\left( {y,t} \right) = Q_{G,1}\left( {y,t} \right) - Q_{D,2}\left( {y,t} \right),$$

11 $$Q_{G,2}\left( {y,t} \right) = Q_{G,1}\left( {y,t} \right) - Q_{D,2}\left( {y,t} \right),$$

where $Q_{I,A}\left( {y,t} \right)$ is the rate of inflow from the Animas River into the San Juan River. We assumed that evaporation and transpiration processes are negligible along the San Juan River. Although there are some return flows from the non-consumptive portion of diversions in SJRB, we omitted them for the sake of tractability.

Confronted with multiple, likely conflicting objectives, it would be impossible to create a single dam release schedule that met all objectives perfectly. Thus, our goal was to find the set of efficient flow designs (i.e., the Pareto frontier) that adhered to dam operation requirements and water availability, and were not strictly outclassed by any other possible flow design^68^. We established this set by maximizing a weighted average *F* of our three objectives and changing the relative weights to obtain designs with different prioritizations^69^:

12 $$F = w_1{\rm{WD}} + w_2{\rm{NF}} + w_3{\rm{NNF}},$$

where *w*_1_, *w*_2_, and *w*_3_ are weights that must sum to one, and the three objectives—human water needs WD, native fish gains NF, and nonnative fish losses NNF—are scaled to lie between zero and one for their worst and best possible values, respectively.

Optimization models and routines were carried out using AIMMS 4.10.2^70^.

### Comparing flow designs and natural flows

We evaluated the strength of the designer flow approach for simultaneously maximizing native and minimizing nonnative fish abundances for each of the three climatic scenarios by comparing flow designs to a natural flow mimicry baseline. For each scenario, we collated the historical, daily inflow into Navajo Reservoir (i.e., upstream from the dam) to ascertain the hypothetical flow patterns of an unregulated San Juan River (Supplementary Fig. 6). We then extracted the total amount of water released during our prescribed three-year flow designs, and distributed that amount per the unregulated inflow patterns to obtain a hypothetical reconstruction of dam operations that would mimic natural flows while fully meeting human water needs. Thus, we could compare the predicted response of native and nonnative fishes to the same volume of water allocated using designer flows versus natural flow mimicry.

### Data availability

Hydrology, water-use, and fish data can be found in the figshare repository: https://figshare.com/s/d7f3524d9fc343d64b63.

## Electronic supplementary material

Supplementary Information

## References

[1] Palmer MA (2008). Climate change and the world’s river basins: anticipating management options. Front. Ecol. Environ..

[2] Rulli MC, Saviori A, D’Odorico P (2013). Global land and water grabbing. Proc. Natl Acad. Sci. USA.

[3] Auerbach DA, Deisenroth DB, McShane RR, McCluney KE, Poff NL (2014). Beyond the concrete: accounting for ecosystem services from free-flowing rivers. Ecosyst. Serv..

[4] Tickner, D. et al. Managing rivers for multiple benefits – a coherent approach to research, policy and planning. *Front. Environ. Sci*. **5**10.3389/fenvs.2017.00004 (2017).

[5] Dyson, M., Bergkamp, G., & Scanlon, J. *Flow: The Essentials of Environmental Flows* (IUCN, Gland, Switzerland, 2003).

[6] Arthington, A. H. *Environmental Flows: Saving Rivers in the Third Millennium* (University of California Press, Berkeley, 2012).

[7] Steffen W, Broadgate W, Deutsch L, Gaffney O, Ludwig C (2015). The trajectory of the Anthropocene: the great acceleration. Anthr. Rev..

[8] Poff NL (1997). The natural flow regime. Bioscience.

[9] Lytle DA, Poff NL (2004). Adaptation to natural flow regimes. Trends Ecol. Evol..

[10] Olden, J. D. in *Conservation of Freshwater Fishes* 107–148 (Cambridge University Press, Cambridge, 2016).

[11] Poff NL, Olden JD, Merritt DM, Pepin DM (2007). Homogenization of regional river dynamics by dams and global biodiversity implications. Proc. Natl Acad. Sci. USA.

[12] Olden JD, Naiman RJ (2010). Incorporating thermal regimes into environmental flows assessments: modifying dam operations to restore freshwater ecosystem integrity. Freshw. Biol..

[13] Acreman M (2014). Environmental flows for natural, hybrid, and novel riverine ecosystems in a changing world. Front. Ecol. Environ..

[14] Yarnell SM (2015). Functional flows in modified riverscapes: Hydrographs, habitats and opportunities. Bioscience.

[15] Poff NL (2015). Sustainable water management under future uncertainty with eco-engineering decision scaling. Nat. Clim. Chang..

[16] Davies PM (2014). Flow-ecology relationships: closing the loop on effective environmental flows. Mar. Freshw. Res..

[17] Bunn SE, Arthington AH (2002). Basic principles and ecological consequences of altered flow regimes for aquatic biodiversity. Environ. Manag..

[18] Cucherousset J, Olden JD (2011). Ecological impacts of nonnative freshwater fishes. Fisheries.

[19] Gido KB, Propst DL, Olden JD, Bestgen KR (2013). Multidecadal responses of native and introduced fishes to natural and altered flow regimes in the American Southwest. Can. J. Fish. Aquat. Sci..

[20] Mims MC, Olden JD (2013). Fish assemblages respond to altered flow regimes via ecological filtering of life history strategies. Freshw. Biol..

[21] Webb JA (2013). Squeezing the most out of existing literature: A systematic re-analysis of published evidence on ecological responses to altered flows. Freshw. Biol..

[22] Keller K, Brown C (2008). Behavioural interactions between the introduced plague minnow *Gambusia holbrooki* and the vulnerable native Australian ornate rainbowfish *Rhadinocentrus ornatus*, under experimental conditions. J. Fish. Biol..

[23] Marks JC, Haden GA, O’Neill M, Pace C (2010). Effects of flow restoration and exotic species removal on recovery of native fish: lessons from a dam decommissioning. Restor. Ecol..

[24] Naiman RJ (2002). Legitimizing fluvial ecosystems as users of water: an overview. Environ. Manag..

[25] Yin X, Yang Z, Petts GE (2012). Optimizing environmental flows below dams. River Res. Appl..

[26] Shiau JT, Wu FC (2013). Optimizing environmental flows for multiple reaches affected by a multipurpose reservoir system in Taiwan: restoring natural flow regimes at multiple temporal scales. Water Resour. Res..

[27] Jager HI, Smith BT (2008). Sustainable reservoir operation: can we generate hydropower and preserve ecosystem values?. River Res. Appl..

[28] Null SE, Lund J (2012). Fish habitat optimization to prioritize river restoration decisions. River Res. Appl..

[29] Richter BD, Mathews R, Harrison DL, Wigington R (2003). Ecologically sustainable water management: managing river flows for ecological integrity. Ecol. Appl..

[30] King J, Louw D (1998). Instream flow assessments for regulated rivers in South Africa using the Building Block Methodology. Aquat. Ecosyst. Health Manag..

[31] Poff NL (2010). The ecological limits of hydrologic alteration (ELOHA): a new framework for developing regional environmental flow standards. Freshw. Biol..

[32] Kiernan JD, Moyle PB, Crain PK (2012). Restoring native fish assemblages to a regulated California stream using the natural flow regime concept. Ecol. Appl..

[33] Olden JD (2014). Are large-scale flow experiments informing the science and management of freshwater ecosystems?. Front. Ecol. Environ..

[34] Franssen NR, Davis JE, Ryden DW, Gido KB (2014). Fish community responses to mechanical removal of nonnative fishes in a large southwestern river. Fisheries.

[35] Valdez RA, Hoffnagle TL, McIvor CC, McKinney T, Leibfried WC (2001). Effects of a test flood on fishes of the Colorado River in Grand Canyon, Arizona. Ecol. Appl..

[36] Gido KB, Propst DL (2012). Long-term dynamics of native and nonnative fishes in the San Juan River, New Mexico and Utah, under a partially managed flow regime. Trans. Am. Fish. Soc..

[37] Rolls RJ (2013). Fish recruitment in rivers with modified discharge depends on the interacting effects of flow and thermal regimes. Freshw. Biol..

[38] Grantham TE, Viers JH, Moyle PB (2014). Systematic screening of dams for environmental flow assessment and implementation. Bioscience.

[39] Rolls RJ, Leigh C, Sheldon F (2012). Mechanistic effects of low-flow hydrology on riverine ecosystems: ecological principles and consequences of alteration. Freshw. Sci..

[40] Walters AW (2016). The importance of context dependence for understanding the effects of low-flow events on fish. Freshw. Sci..

[41] Jaeger KL, Olden JD, Pelland NA (2014). Climate change poised to threaten hydrologic connectivity and endemic fishes in dryland streams. Proc. Natl Acad. Sci. USA.

[42] Melis TS, Walters CJ, Korman J (2015). Surprise and opportunity for learning in Grand Canyon: the Glen Canyon dam adaptive management program. Ecol. Soc..

[43] Shafroth PB (2010). Ecosystem effects of environmental flows: modelling and experimental floods in a dryland river. Freshw. Biol..

[44] Robinson CT (2012). Long-term changes in community assembly, resistance, and resilience following experimental floods. Ecol. Appl..

[45] Cross WF (2011). Ecosystem ecology meets adaptive management: food web response to a controlled flood on the Colorado River, Glen Canyon. Ecol. Appl..

[46] Rheinheimer DE, Null SE, Lund JR (2014). Optimizing selective withdrawal from reservoirs to manage downstream temperatures with climate warming. J. Water Resour. Plan. Manag..

[47] Khan NM, Tingsanchali T (2009). Optimization and simulation of reservoir operation with sediment evacuation: a case study of the Tarbela Dam, Pakistan. Hydrol. Process..

[48] Ziv G, Baran E, Nam S, Rodríguez-Iturbe I, Levin SA (2012). Trading-off fish biodiversity, food security, and hydropower in the Mekong River Basin. Proc. Natl Acad. Sci. USA.

[49] Zarfl C, Lumsdon AE, Berlekamp J, Tydecks L, Tockner K (2015). A global boom in hydropower dam construction. Aquat. Sci..

[50] Null SE, Ligare ST, Viers JH (2013). A method to consider whether dams mitigate climate change effects on stream temperatures. J. Am. Water Resour. Assoc..

[51] Deemer BR (2016). Greenhouse gas emissions from reservoir water surfaces: a new global synthesis. Bioscience.

[52] Stewart-Koster B, Olden JD, Gido KB (2014). Quantifying flow-ecology relationships with functional linear models. Hydrol. Sci. J..

[53] Rosenfeld JS (2017). Developing flow-ecology relationships: Implications of nonlinear biological responses for water management. Freshw. Biol..

[54] Wagener T (2010). The future of hydrology: an evolving science for a changing world. Water Resour. Res..

[55] Kennard MJ, Olden JD, Arthington AH, Pusey BJ, Poff NL (2007). Multiscale effects of flow regime and habitat and their interaction on fish assemblage structure in eastern Australia. Can. J. Fish. Aquat. Sci..

[56] Speed, R., et al. *River restoration: a strategic approach to planning and management* (UNESCO, Paris, 2016).

[57] Jackson S, Pollino C, Maclean K, Bark R, Moggridge B (2015). Meeting Indigenous peoples’ objectives in environmental flow assessments: case studies from an Australian multi-jurisdictional water sharing initiative. J. Hydrol..

[58] Horne A (2016). Optimization tools for environmental water decisions: a review of strengths, weaknesses, and opportunities to improve adoption. Environ. Model. Softw..

[59] Propst DL, Gido KB (2004). Responses of native and nonnative fishes to natural flow regime mimicry in the San Juan River. Trans. Am. Fish. Soc..

[60] Ruhi A, Olden JD, Sabo JL (2016). Declining streamflow induces collapse and replacement of native fish in the American Southwest. Front. Ecol. Environ..

[61] Leonard Rice Engineering. *San Juan and Dolores River Basin Information*. Available at: http://cwcbweblink.state.co.us/WebLink/0/doc/125269/Page1.aspx (2005)

[62] Gido KB, Propst DL (1999). Habitat use and association of native and nonnative fishes in the San Juan River, New Mexico and Utah. Copeia.

[63] Hines, B. *Endangered Fish Monitoring and Nonnative Fish Control in the Lower San Juan River 2014* (Utah Division of Wildlife Resources, Moab, Utah, 2015).

[64] Yen JD, Thomson JR, Paganin DM, Keith JM, Mac Nally R (2015). Function regression in ecology and evolution: FREE. Methods Ecol. Evol..

[65] James GM, Wang J, Zhu J (2009). Functional linear regression that’s interpretable. Ann. Stat..

[66] Chen, W. & Olden, J. D. Evaluating transferability of flow-ecology relationships across space, time, and taxonomy. *Freshw. Biol.*10.1111/fwb.13041 (2017).

[67] Holden, P. Flow recommendations for the San Juan River. Available at: https://www.fws.gov/southwest/sjrip/pdf/DOC_Flow_recommendations_San_Juan_River.pdf (1999).

[68] Deb K. in *Search Methodologies* (eds Burke, E. K. & Kendall, G.) 403–449 (Springer, New York, 2014).

[69] Tóth SF, McDill ME (2009). Finding efficient harvest schedules under three conflicting objectives. Ann. For. Sci..

[70] Bisschop, J. *AIMMS Optimization Modeling*. Available at: http://download.aimms.com/aimms/download/manuals/AIMMS3_OM.pdf (2016).

