# Supplementary material for: Designing flows to resolve human and environmental water needs in a dam-regulated river
Source: Nat Commun. 2017 Dec 18;8:2158. doi: 10.1038/s41467-017-02226-4 (PMC5735146; doi:10.1038/s41467-017-02226-4)
Supplement: Supplementary file 1 — Supplementary Information [file 41467_2017_2226_MOESM1_ESM.pdf]

SUPPLEMENTARY FIGURES

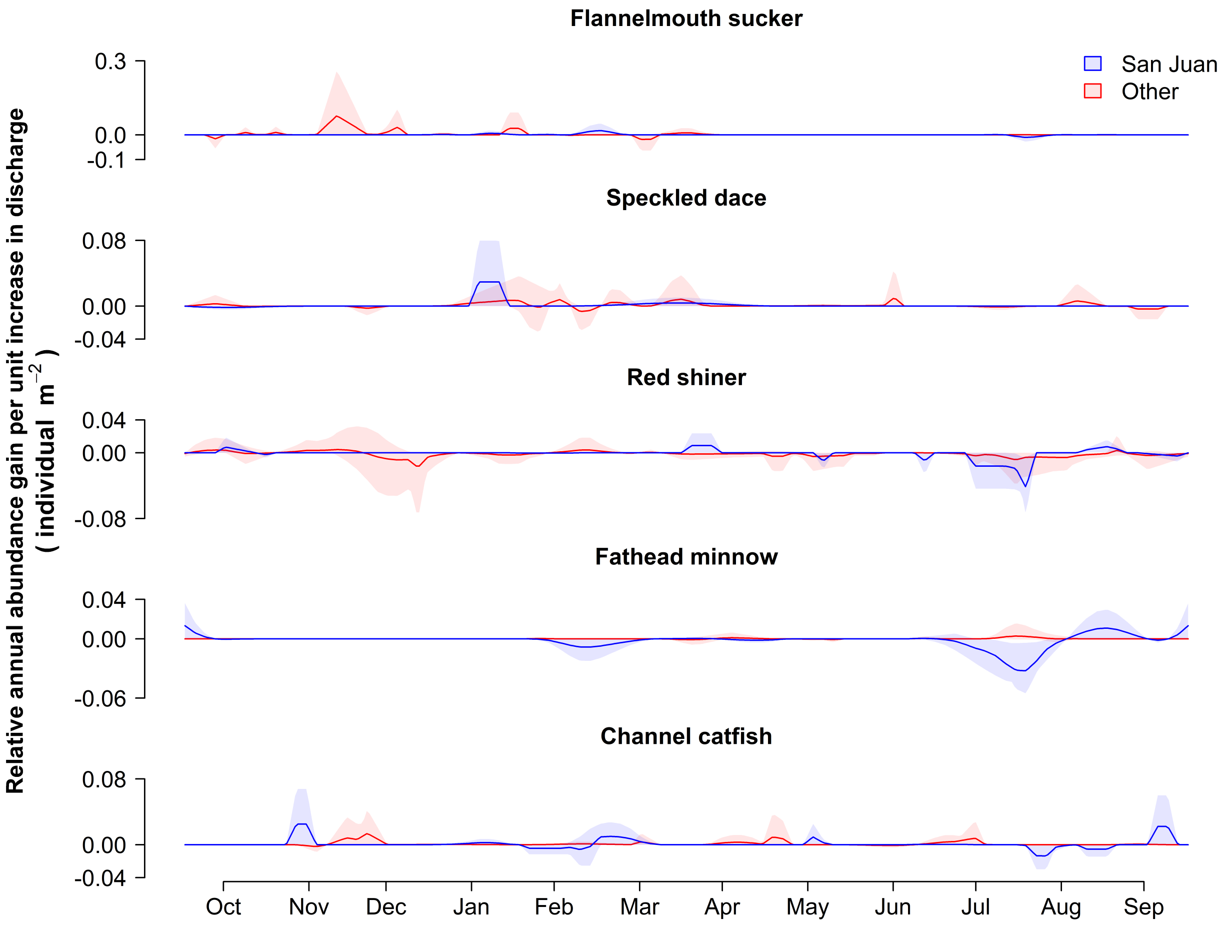

Supplementary Figure 1. Comparison of flow-ecology relationships (functional regression coefficients) derived for the San Juan River (blue) versus those for the Gila, Pecos, San Pedro, and Virgin Rivers (red). Lines represent average response across all flow-ecology models while shading encompasses the 90% confidence interval.

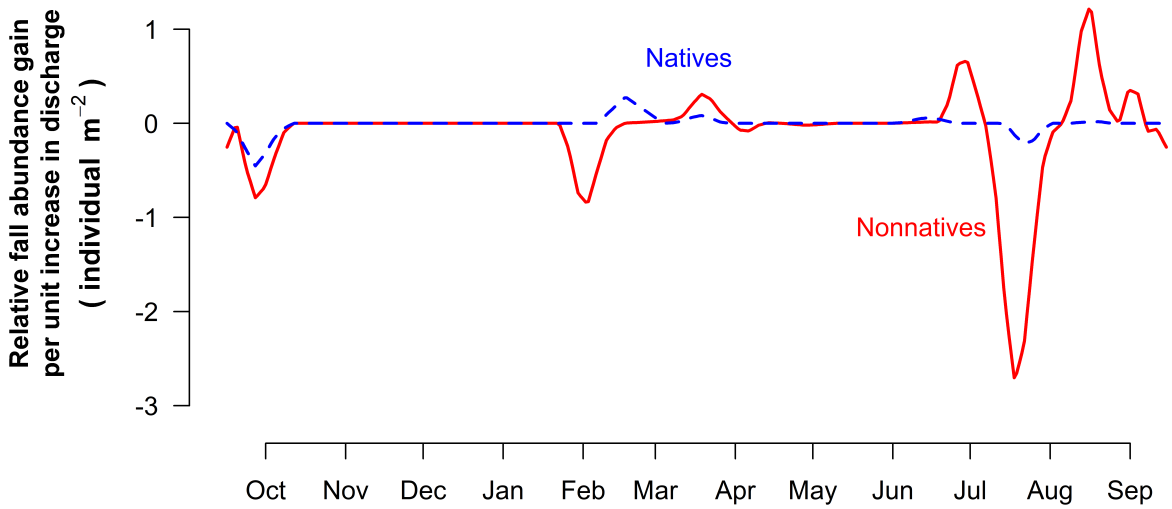

Supplementary Figure 2. Daily response of native and nonnative fish assemblages per unit increase in daily log-transformed discharge aggregated from flow-ecology models. Aggregated natives' (blue, dashed line) and nonnatives' (red, solid line) relative fall abundance response per unit increase in log-transformed discharge on each day of an antecedent year. Results derived from flow-ecology functional regression models.

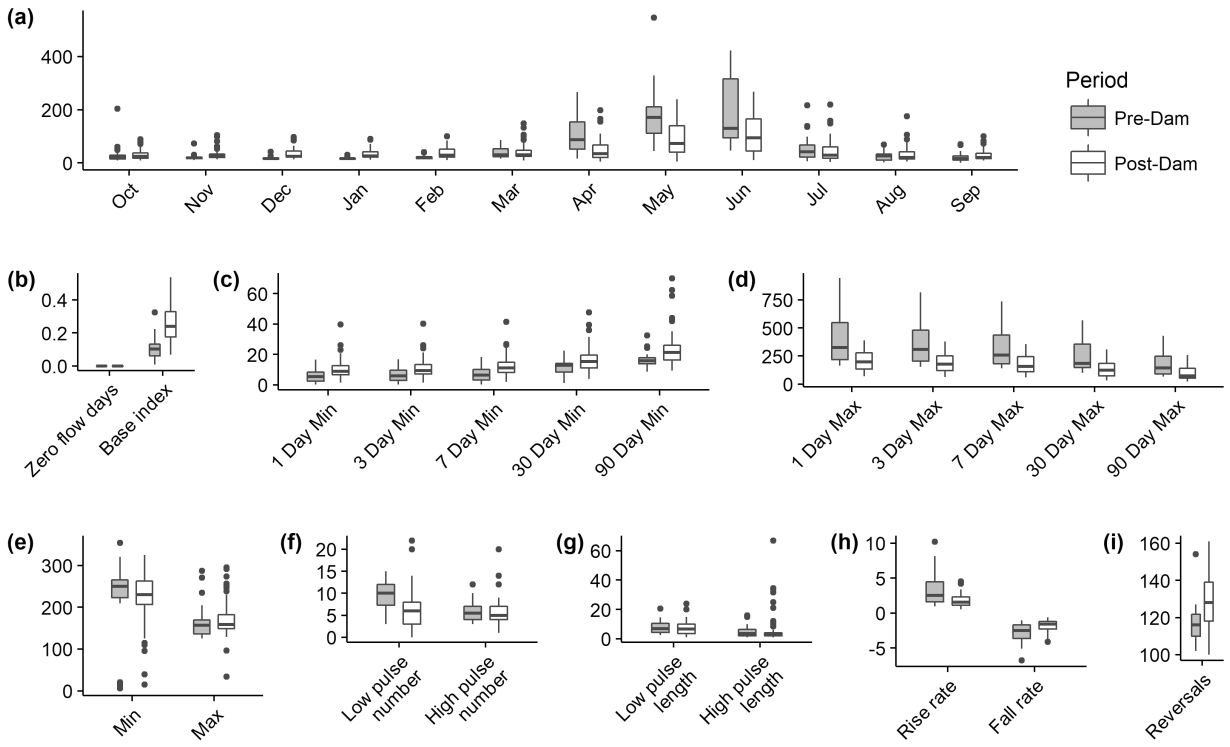

Supplementary Figure 3. San Juan River hydrologic regimes before (1935-1956) and after (1963-2015) the construction of Navajo Dam. Comparison of San Juan River hydrologic regimes at the USGS flow gage station 09368000 near Shiprock, NM before (pre-dam) and after (post-dam) the construction of the Navajo Dam, using Indicators of Hydrologic Alteration[1]. (a) Mean monthly discharges [m^3 s^{-1}]; (b) Base flow conditions; (c) Minimum flow conditions [m^3 s^{-1}]; (d) Maximum flow conditions [m^3 s^{-1}]; (e) Timing of minimum and maximum discharge [calendar day]; (f) Number of flood pulses; (g) Length of flood pulses [days]; (h) Mean of rising and falling discharge rates [m^3 s^{-1} day^{-1}]; (i) Number of reversals between rising and falling daily discharge. *W*hiskers *represent* 1.5*IQR (inner quartile range), *and points are* outliers outside of that range. Discharge was similar at USGS flow gage stations near Four Corners, CO and Bluff, Utah.

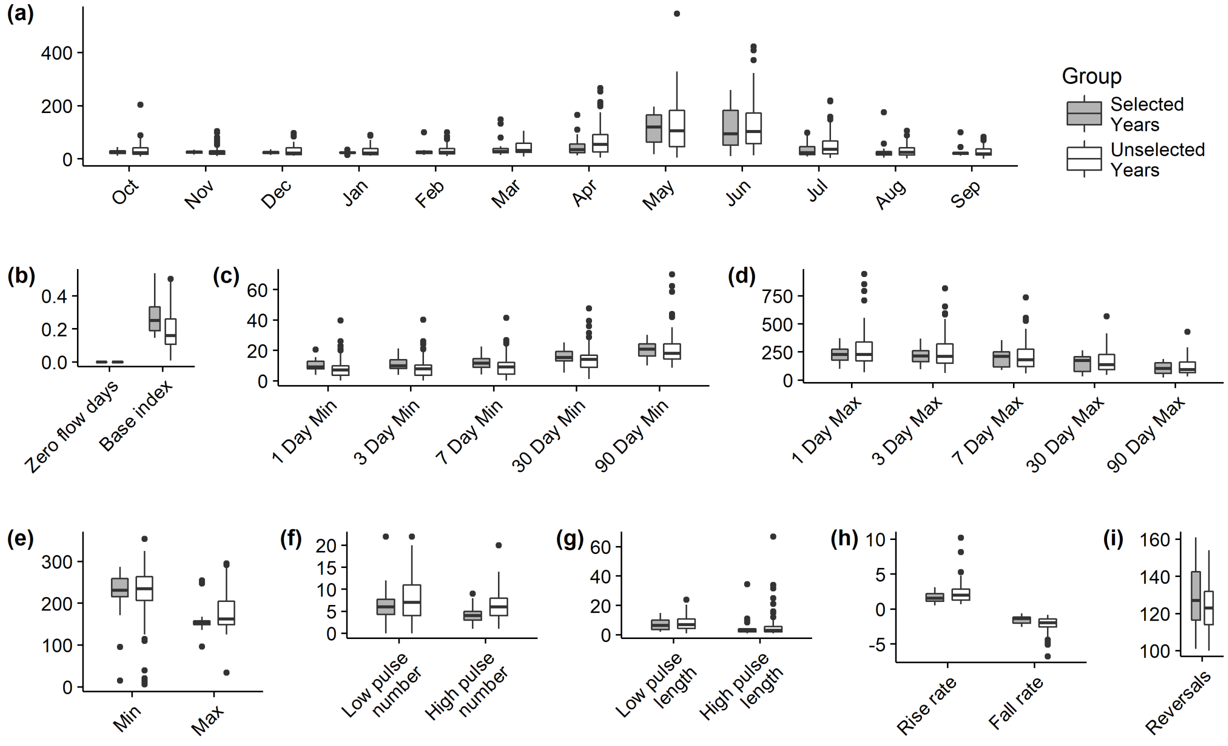

Supplementary Figure 4. San Juan River post-dam hydrologic regimes in study period (1993-2010) and outside of the study period (1963-1992, 2011-2015) after dam construction. Comparison of San Juan River hydrologic regimes at the USGS flow gage station 09368000 near Shiprock, NM within and outside our study period (Selected and Unselected Years, respectively), using Indicators of Hydrologic Alteration[1]. (a) Mean monthly discharges [m^3 s^{-1}]; (b) Base flow conditions; (c) Minimum flow conditions [m^3 s^{-1}]; (d) Maximum flow conditions [m^3 s^{-1}]; (e) Timing of minimum and maximum discharge [calendar day]; (f) Number of flood pulses; (g) Length of flood pulses [days]; (h) Mean of rising and falling discharge rates [m^3 s^{-1} day^{-1}]; (i) Number of reversals between rising and falling daily discharge. Whiskers represent 1.5*IQR (inner quartile range), and points are outliers outside of that range. Discharge was similar at USGS flow gage stations near Four Corners, CO and Bluff, Utah.

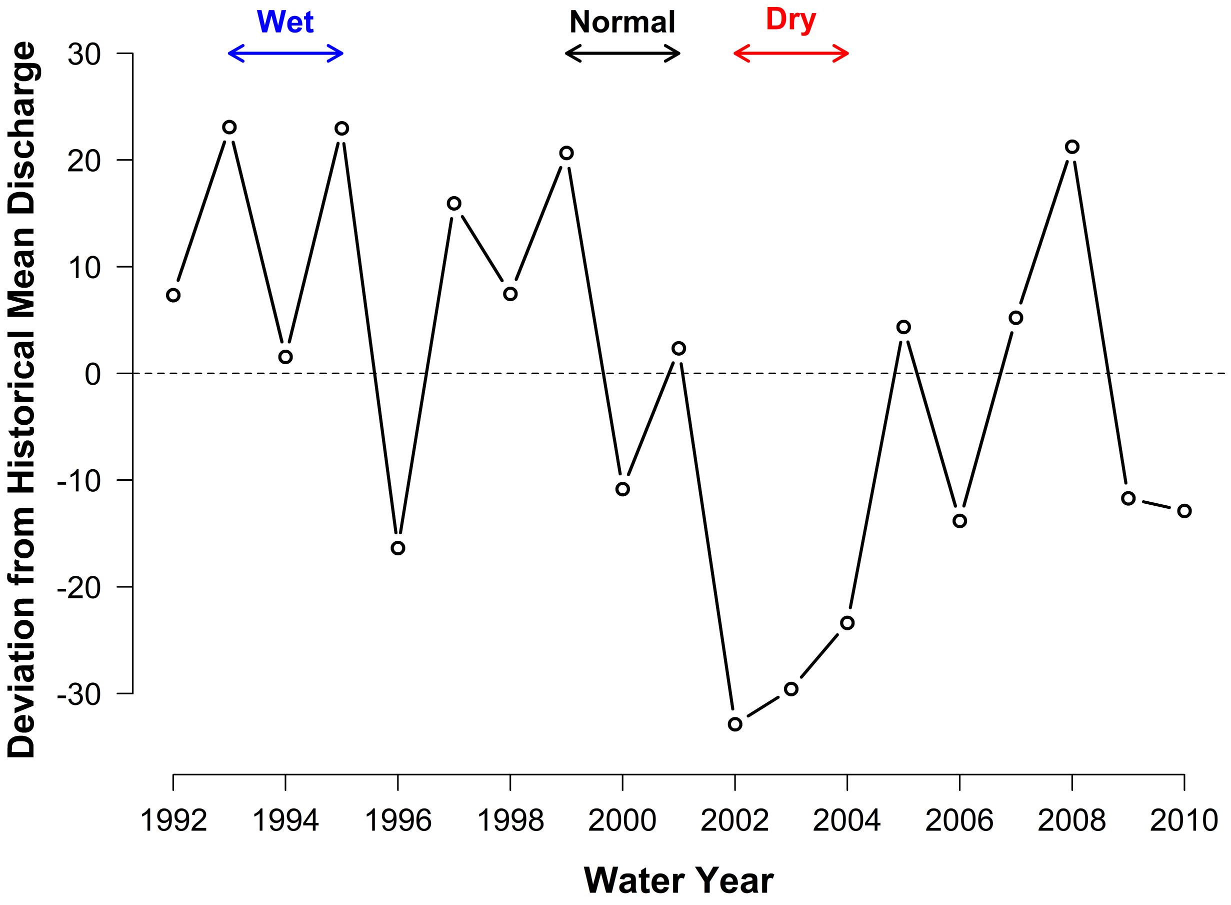

Supplementary Figure 5. Annual river discharge anomalies and selected climatic scenarios. River discharge anomalies from historical seasonal trend for water years 1992 through 2010 (based on USGS flow gage station 09368000 near Shiprock, NM). Climatic scenarios representing wet, normal, and dry flow periods are characterized by positive, minimal, and negative anomalies, respectively.

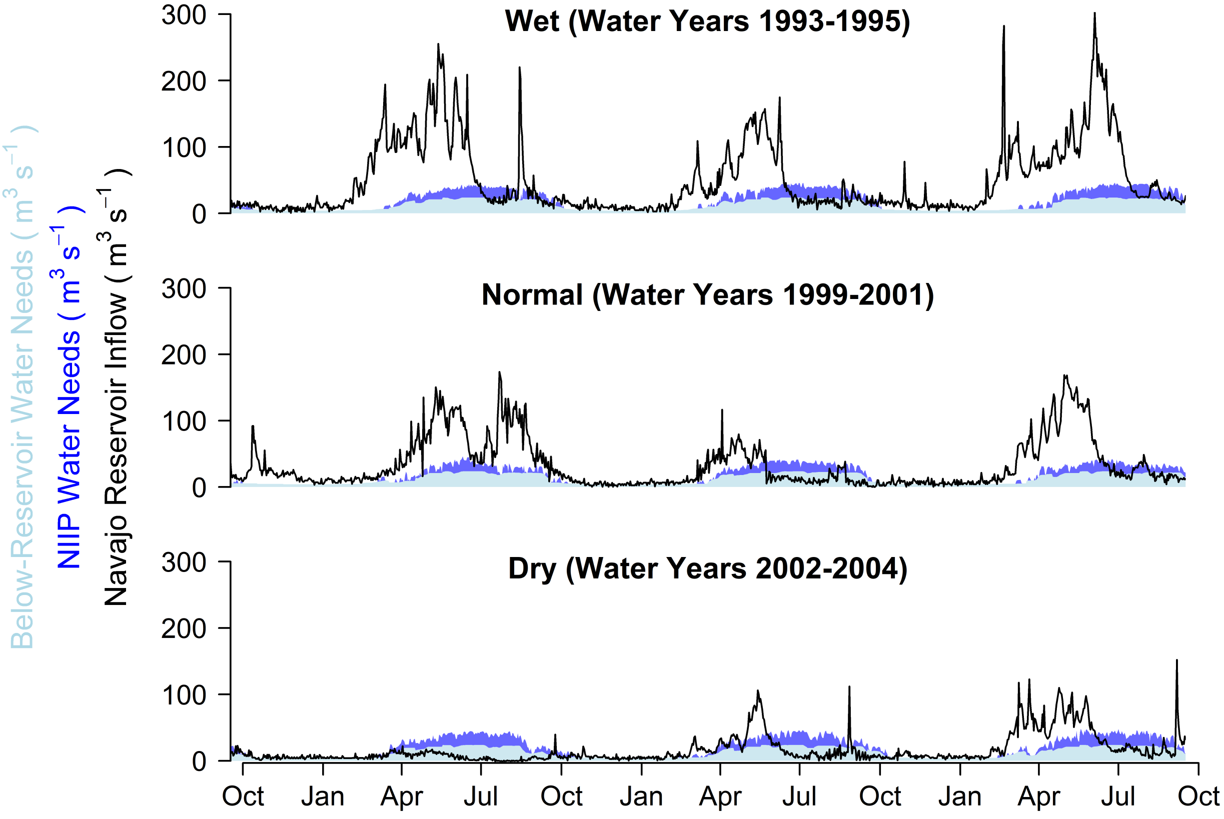

Supplementary Figure 6. Reservoir inflows and water diversion needs for the San Juan River Basin. Daily inflow into Navajo Reservoir (solid line), water diversion demands from the Navajo Indian Irrigation Project (blue fill), and water diversion demands from downstream of Navajo Reservoir (light blue fill) during each of the three climatic scenarios. The patterns of inflow were scaled to match the total amount of water used in optimized flow designs when simulating a hypothetical natural flow mimicry approach in the San Juan River.

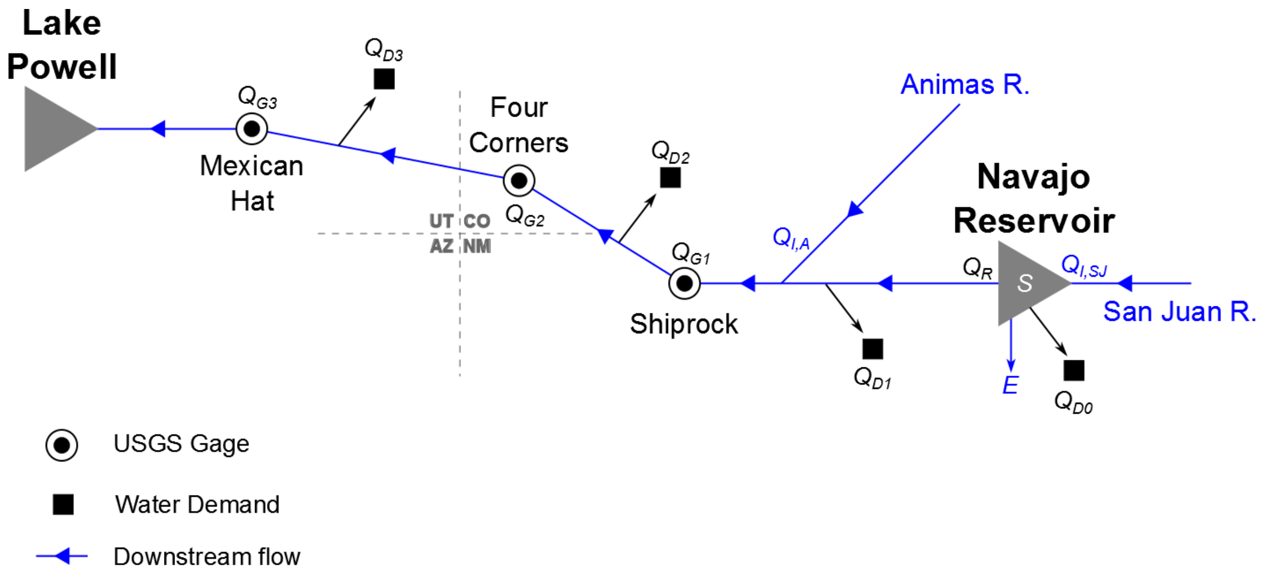

Supplementary Figure 7. Schematic of San Juan River operating model. Schematic of the San Juan River depicting water inflows, depletions and demands (described in Supplementary Table 2) that were included in the optimization framework. Black lettering signifies decision variables to optimize over while blue lettering signifies known values. Map not drawn to scale.

SUPPLEMENTARY TABLES

Supplementary Table 1. Model diagnostics of selected flow-ecology model for each species and USGS gage combination.

Species	USGS Gage	Median CV Error*		NRMSE †	R^2
		Min	Max		
Native					
Catostomus latipinnis flannelmouth sucker	Mexican Hat	20.90	44.04	0.23	0.04
	Four Corners	22.08	64.66	0.20	0.27
	Shiprock	26.02	49.28	0.17	0.50
Rhinichthys osculus speckled dace	Mexican Hat	8.22	16.35	0.29	0
	Four Corners	13.73	20.58	0.26	0
	Shiprock	13.50	20.95	0.27	0.42
Nonnative					
Cyprinella lutrensis red shiner	Mexican Hat	53.06	87.43	0.19	0.55
	Four Corners	23.95	50.19	0.19	0.50
	Shiprock	79.60	149.86	0.14	0.62
Ictalurus punctatus channel catfish	Mexican Hat	25.06	38.43	0.13	0.58
	Four Corners	10.55	17.60	0.26	0
	Shiprock	23.82	47.63	0.28	0

Pimephales promelas fathead minnow	Mexican Hat	89.89	153.36	0.16	0.70
	Four Corners	77.66	197.17	0.25	0.34
	Shiprock	64.95	122.26	0.13	0.71

* Minimum median cross-validation error was used to select models; maximum median cross-validation errors were derived from all considered models

† Root-mean squared error, normalized by the range of the response, log-transformed species density

Supplementary Table 2. Summary of historical data within each climatic scenario.

	Period†		
	Wet	Normal	Dry
Characteristic	1993-1995	1999-2001	2002-2004
Hydrology Totals			
Reservoir Inflow (km^3)	4.782	3.142	1.536
Reservoir Evaporation (km^3)	0.113	0.100	0.073
Animas River Inflow (km^3)	2.859	2.276	1.206
Precipitation (mm)*	1013	877	797
Reservoir Operations			
Initial Reservoir Capacity (km^3)	1.947	1.705	1.737
Final Reservoir Capacity (km^3)	1.919	1.738	1.153
Dam Release Total (km^3)	4.043	2.515	1.360
Water Diversion Totals			
Navajo Reservoir (km^3)	0.656	0.491	0.688
Downstream San Juan (km^3)	1.191	1.178	1.190

*Precipitation data collated from PRISM Climate Group. All other data collated from United States Bureau of Reclamation.

†Periods are based on water years (October - September)

Supplementary Table 3. Description [and units] of parameters and variables used in optimization model.

Known inflows, depletions, and demands	
$Q_{I,SJ}$	Inflow of water from the San Juan River to Navajo Reservoir [m^3 s^{-1}]
$Q_{I,A}$	Inflow of water from the Animas River to the San Juan River [m^3 s^{-1}]
E	Daily volume of water evaporation from the Navajo Reservoir [m^3]
d_0	Flow required by the Navajo Indian Irrigation Project [m^3 s^{-1}]
d_1	Flow required by users downstream of Navajo Reservoir and upstream of the USGS gage near Shiprock, NM [m^3 s^{-1}]
d_2	Flow required by users downstream of the Shiprock gage and above the USGS gage near Four Corners, CO [m^3 s^{-1}]
d_3	Flow required by users downstream of the Four Corners gage and above the USGS gage near Mexican Hat, UT [m^3 s^{-1}]
Known reservoir capacities and requirements	
S_{min}	Minimum volume of water that must be stored in Navajo Reservoir for it to be active [m^3]
S_{max}	Maximum volume of water that the Navajo Reservoir can hold [m^3]
$Q_{R,min}$	Minimum release flow from the Navajo Reservoir required by the US Bureau of Reclamation [m^3 s^{-1}]
$Q_{R,max}$	Maximum release flow through the Navajo Dam outlet [m^3 s^{-1}]
$\delta_{R,max}$	Maximum absolute amount that Navajo releases can change by per time step [m^3 s^{-1}]
γ	Conversion factor between flow in m^3 s^{-1} to volume per day in m^3
Decision Variables	
Q_R	Flow released through the Navajo Dam [m^3 s^{-1}]
$Q_{D,0}$	Flow diverted from Navajo Reservoir for the Navajo Indian Irrigation Project [m^3 s^{-1}]
$Q_{D,1}$	Flow diverted between Navajo Reservoir and the USGS gage near Shiprock, NM [m^3 s^{-1}]
$Q_{D,2}$	Flow diverted between the Shiprock and Four Corners USGS gages [m^3 s^{-1}]
$Q_{D,3}$	Flow diverted between the Four Corners and Mexican Hat USGS gages [m^3 s^{-1}]
$Q_{G,1}$	Discharge at the USGS gage near Shiprock [m^3/ s^{-1}]
$Q_{G,2}$	Discharge at the Four Corners USGS gage [m^3 s^{-1}]
$Q_{G,3}$	Discharge at the Mexican Hat USGS gage [m^3 s^{-1}]

Supplementary References

1. Richter, B. D., Baumgartner, J. V., Powell, J. & Braun, D. P. A method for assessing hydrologic alteration within ecosystems. *Conserv. Biol.* **10,** 1163-1174 (1996).
